# Diabetes Duration Is Associated with Declining Kidney Function: eGFR and CKD Burden Across Duration

**DOI:** 10.3390/jcm15062235

**Published:** 2026-03-15

**Authors:** Carmen Pantis, Cosmin Mihai Vesa, Timea Claudia Ghitea, Daniela Florina Trifan, Roxana Daniela Brata, Nicolae Ovidiu Pop, Madalina Ioana Moisi

**Affiliations:** 1 Clinical County Emergency Hospital Bihor, 410169 Oradea, Romania; carmen.pantis@didactic.uoradea.ro; 2 Doctoral School of Biomedical Sciences, Faculty of Medicine and Pharmacy, University of Oradea, 410087 Oradea, Romania; 3 Department of Preclinical Discipline, Faculty of Medicine and Pharmacy, University of Oradea, 410068 Oradea, Romania; pop.nicolaeovidiu@didactic.uoradea.ro (N.O.P.);; 4 Pharmacy Department, Faculty of Medicine and Pharmacy, University of Oradea, 410068 Oradea, Romania; 5 Department of Clinical Discipline, Faculty of Medicine and Pharmacy, University of Oradea, 410068 Oradea, Romania; trifan.daniela17@yahoo.com (D.F.T.); brata.roxanadaniela@didactic.uoradea.ro (R.D.B.)

**Keywords:** type 2 diabetes mellitus, diabetes duration, chronic kidney disease, CKD, eGFR, diabetic kidney disease, renal decline, microvascular complications, hypertension, metabolic exposure

## Abstract

**Background:** Diabetic kidney disease is a major complication of type 2 diabetes mellitus (T2DM) and a leading cause of chronic kidney disease (CKD) worldwide. While diabetes duration is often considered a marker of cumulative metabolic exposure, its independent contribution to renal decline beyond aging and hypertension remains incompletely defined. **Methods:** We conducted a cross-sectional study including 250 adults with T2DM. Diabetes duration was analyzed both as a continuous variable and across four predefined strata (0–4, 5–9, 10–14, and ≥15 years). The primary endpoint was estimated glomerular filtration rate (eGFR), analyzed as a continuous outcome. Functional CKD was defined as eGFR < 60 mL/min/1.73 m^2^. Linear and logistic regression models were constructed in unadjusted and adjusted forms (age, sex, BMI, hypertension, HbA1c). A sensitivity analysis modeling duration per 5-year increase was performed. **Results:** Mean eGFR declined significantly across duration strata (82.45, 84.27, 78.72, and 61.57 mL/min/1.73 m^2^, respectively; *p* < 0.001). The prevalence of functional CKD increased markedly in patients with ≥15 years of diabetes (54.2%) compared with shorter-duration groups (~15–18%; *p* < 0.001). In linear regression, each additional year of diabetes was associated with a 1.32 mL/min/1.73 m^2^ decline in eGFR (*p* < 0.001), remaining significant after adjustment (β = −0.85; *p* < 0.001). In logistic regression, each additional year was associated with a 10.7% increase in adjusted odds of CKD (OR = 1.11; 95% CI 1.04–1.17; *p* < 0.001). Each 5-year increment conferred a 66% increase in adjusted CKD risk (OR = 1.66; 95% CI 1.25–2.21; *p* < 0.001). Patients with ≥15 years of diabetes had nearly fourfold higher adjusted odds of CKD compared with those with 0–4 years (OR = 3.90; 95% CI 1.42–10.75; *p* = 0.008). **Conclusions:** Diabetes duration is strongly and independently associated with declining kidney function. Prolonged disease exposure confers a substantial increase in CKD risk, even after adjustment for age, hypertension, and metabolic factors. These findings highlight the progressive nephrotoxic impact of cumulative hyperglycemic exposure and underscore the need for early and sustained nephroprotective strategies in T2DM.

## 1. Introduction

Type 2 diabetes mellitus (T2DM) is a leading cause of chronic kidney disease (CKD) worldwide and represents the most common etiology of end-stage renal disease (ESRD). Diabetic kidney disease (DKD) is a major contributor to morbidity, mortality, and healthcare burden, significantly increasing the risk of cardiovascular events and premature death. Despite advances in glycemic management and nephroprotective therapies, CKD remains highly prevalent among individuals with long-standing diabetes [1,2,3,4]. In Romania, the burden of diabetes is substantial. According to data from the International Diabetes Federation and national epidemiological reports, the prevalence of diabetes among adults is estimated at approximately 11–12%, placing Romania among the European countries with a high diabetes burden. Furthermore, a significant proportion of cases remain undiagnosed, contributing to delayed detection of complications such as chronic kidney disease [5].

The development of diabetic kidney disease is driven by complex and interrelated mechanisms, including chronic hyperglycemia, glomerular hyperfiltration, oxidative stress, activation of the renin–angiotensin–aldosterone system, low-grade inflammation, and progressive microvascular injury. Over time, sustained metabolic stress leads to mesangial expansion, thickening of the glomerular basement membrane, podocyte loss, and nephron depletion, culminating in an irreversible decline in glomerular filtration rate (GFR) [6,7,8].

Diabetes duration is commonly considered a surrogate marker of cumulative metabolic exposure and is often associated with the risk of microvascular complications. However, the independent contribution of disease duration to renal impairment remains difficult to disentangle from the effects of aging, hypertension, obesity, and glycemic control. While chronological aging is known to be associated with a gradual physiological decline in GFR, it remains unclear to what extent prolonged diabetes exposure accelerates renal dysfunction beyond age-related changes [9,10,11,12].

Clinical observations suggest that early kidney injury may already be present at the time of diabetes diagnosis, reflecting years of subclinical metabolic dysfunction prior to formal detection. Nevertheless, the magnitude and pattern of renal decline across different diabetes duration strata have not been consistently characterized in real-world populations, particularly in Eastern European cohorts [13,14,15].

Furthermore, it is important to determine whether renal impairment exhibits a linear relationship with diabetes duration or whether a threshold effect emerges after prolonged disease exposure. Identifying clinically meaningful temporal patterns may improve risk stratification and guide early nephroprotective interventions.

Therefore, the aim of the present study was to investigate the association between diabetes duration and kidney function in a real-world cohort of adults with T2DM. Specifically, we sought to (1) evaluate differences in estimated glomerular filtration rate (eGFR) across predefined duration strata; (2) assess the association between diabetes duration and functional CKD (eGFR < 60 mL/min/1.73 m^2^); and (3) determine whether these associations persist after adjustment for age, hypertension, and metabolic covariates.

## 2. Materials and Methods

### 2.1. Study Design and Participants

This study represents a cross-sectional analysis conducted in adult patients with type 2 diabetes mellitus. Participants were consecutively recruited from the outpatient diabetes and internal medicine clinics of the Bihor County Emergency Clinical Hospital in Oradea, Romania. The sampling base therefore consisted of adult patients with established T2DM receiving routine clinical follow-up at this tertiary care center.

Eligible participants were adults aged 18–80 years with a confirmed diagnosis of T2DM according to American Diabetes Association (ADA) criteria. All patients underwent standardized clinical and laboratory evaluation between March 2024 and September 2025. Patients with incomplete records regarding diabetes duration or kidney function parameters were excluded. Individuals with acute kidney injury at the time of assessment were also excluded to ensure evaluation of stable renal function.

Initially, 274 patients with type 2 diabetes mellitus were screened for eligibility during the study period. Among these, 24 individuals were excluded due to incomplete data regarding diabetes duration or renal function parameters (*n* = 18) or due to the presence of acute kidney injury at the time of evaluation (*n* = 6). Consequently, a total of 250 participants with complete clinical and laboratory data were included in the final analysis. All participants provided informed consent for the use of anonymized clinical data for research purposes.

The study was approved by the Ethics Committee of the Faculty of Medicine and Pharmacy, University of Oradea (approval number 5/30 October 2023).

### 2.2. Clinical and Renal Assessment

Diabetes duration was determined based on the documented date of diagnosis recorded in the medical records. Duration was calculated as the interval between the recorded diagnosis of type 2 diabetes mellitus and the date of the clinical evaluation included in the present study. For categorical analyses, diabetes duration was stratified into four groups: 0–4 years, 5–9 years, 10–14 years, and ≥15 years. Antidiabetic and antihypertensive treatments were heterogeneous and reflected routine clinical management. Although detailed pharmacological stratification was not included in the regression models, hypertension status was incorporated as a covariate, partially accounting for the use of renin–angiotensin system blockers commonly prescribed in this population.

The primary endpoint was estimated glomerular filtration rate (eGFR; RFG), analyzed as a continuous variable. eGFR was calculated using the CKD-EPI equation and expressed in mL/min/1.73 m^2^.

Functional chronic kidney disease (CKD) was defined as eGFR < 60 mL/min/1.73 m^2^ and analyzed as a binary outcome.

Secondary renal parameters included serum creatinine and urea levels. When available, a documented clinical diagnosis of chronic kidney disease (Boalacronicaderinichi) was recorded for descriptive purposes.

Covariates included age (years), sex, body mass index (BMI, kg/m^2^), hypertension status (HTA1DA), and glycated hemoglobin (HbA1c, %). HbA1c values corresponded to measurements obtained at the time of the clinical evaluation included in the analysis. Historical HbA1c data were not consistently available for all participants and were therefore not used to estimate cumulative glycemic burden.

### 2.3. Anthropometric and Laboratory Measurements

Body weight and height were measured using calibrated scales, and BMI was calculated as weight (kg) divided by height squared (m^2^).

Blood pressure was measured in the seated position after at least 5 min of rest using a validated automated sphygmomanometer.

Venous blood samples were obtained after overnight fasting. Serum creatinine, urea, and fasting glucose were measured using standardized enzymatic methods in an accredited laboratory. HbA1c was determined using high-performance liquid chromatography (HPLC). eGFR was calculated using the CKD-EPI formula.

All measurements were performed under standardized clinical conditions.

### 2.4. Statistical Analysis

Continuous variables were primarily expressed as mean ± standard deviation (SD). In addition, median values and interquartile ranges (IQR) were examined when appropriate to evaluate the distribution of continuous variables.

Comparisons of eGFR across diabetes duration strata were performed using one-way analysis of variance (ANOVA). When appropriate, median values were also examined to assess distributional consistency. The association between duration strata and CKD prevalence (eGFR < 60 mL/min/1.73 m^2^) was evaluated using the chi-square test. When the ANOVA test indicated significant differences, post hoc comparisons were performed using the Tukey honestly significant difference (HSD) test to identify pairwise differences between duration groups.

Linear regression models were constructed to assess the association between diabetes duration and eGFR. Diabetes duration was analyzed as:

1. A continuous variable (per 1-year increase),
2. A clinically interpretable continuous variable (per 5-year increase),
3. A categorical variable (≥15 years vs 0–4 years).

Multivariable linear regression models were adjusted for age, sex, BMI, hypertension status, and HbA1c.

Logistic regression analyses were performed using functional CKD (eGFR < 60 mL/min/1.73 m^2^) as the dependent variable. Both unadjusted and adjusted models were constructed. Adjusted models included age, sex, BMI, hypertension status, and HbA1c as covariates.

Odds ratios (OR) and 95% confidence intervals (CI) were calculated for logistic regression models, and beta coefficients (β) with 95% CI were reported for linear regression models.

All statistical tests were two-tailed, and statistical significance was defined as *p* < 0.05. Statistical analyses were performed using IBM SPSS Statistics, Version 30 (IBM Corp., Armonk, NY, USA).

### 2.5. Sample Size Considerations

This analysis included 250 patients with complete renal and clinical data. Given the observational design, all eligible consecutive patients were included to maximize statistical power.

For logistic regression models evaluating functional CKD, the number of outcome events exceeded the conventional threshold of 10 events per variable (EPV), which is generally recommended to ensure adequate model stability and minimize the risk of overfitting [16]. The distribution across duration strata (*n* = 52, 54, 96, and 48, respectively) provided sufficient power to detect clinically meaningful differences in eGFR and CKD prevalence, particularly in comparisons involving long-standing diabetes.

### 2.6. Ethical Considerations

This study was observational and involved analysis of routinely collected clinical data. No experimental interventions were performed. Data were anonymized prior to analysis to ensure participant confidentiality.

## 3. Results

### 3.1. Kidney Function Across Diabetes Duration Strata

A total of 250 adults with type 2 diabetes were stratified according to diabetes duration into four predefined categories: 0–4 years (*n* = 52), 5–9 years (*n* = 54), 10–14 years (*n* = 96), and ≥15 years (*n* = 48). Kidney function, assessed by estimated glomerular filtration rate (eGFR), differed significantly across duration strata.

Mean eGFR progressively declined with increasing diabetes duration. Patients with 0–4 years of diabetes had a mean eGFR of 82.45 ± 24.54 mL/min/1.73 m^2^, while those with 5–9 years had a mean value of 84.27 ± 22.34 mL/min/1.73 m^2^. In the 10–14-year group, mean eGFR decreased to 78.72 ± 21.46 mL/min/1.73 m^2^. Notably, patients with ≥15 years of diabetes exhibited a marked reduction in renal function, with a mean eGFR of 61.57 ± 22.38 mL/min/1.73 m^2^. One-way analysis of variance (ANOVA) confirmed a highly significant difference across groups (*p* < 0.001).

Median eGFR values followed a similar pattern (83.17, 87.76, 80.99, and 58.15 mL/min/1.73 m^2^, respectively), further supporting a clinically meaningful decline in renal function among patients with long-standing diabetes.

When functional chronic kidney disease (CKD) was defined as eGFR < 60 mL/min/1.73 m^2^, its prevalence increased substantially with longer diabetes duration. The proportion of patients meeting CKD criteria was 17.3% in the 0–4-year group, 14.8% in the 5–9-year group, and 17.7% in the 10–14-year group. In contrast, 54.2% of patients with ≥15 years of diabetes had eGFR < 60 mL/min/1.73 m^2^. The association between diabetes duration strata and CKD prevalence was highly statistically significant (chi-square test, *p* < 0.001).

Overall, these findings indicate a pronounced deterioration in kidney function after prolonged diabetes exposure, with a particularly sharp increase in CKD burden in patients with disease duration exceeding 15 years (Table 1).

**Figure 1:**
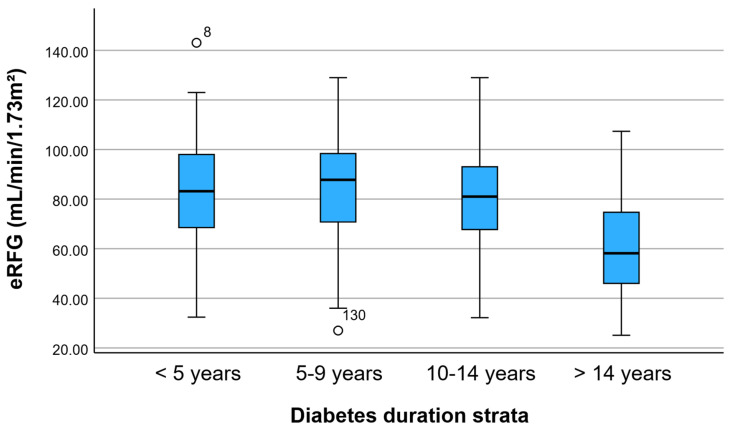
eGFR across diabetes duration strata. Boxplot illustrating the distribution of estimated glomerular filtration rate (eGFR, mL/min/1.73 m^2^) across diabetes duration categories (0–4, 5–9, 10–14, and ≥15 years). A marked downward shift in eGFR is observed in the ≥15-year group, consistent with a substantially higher renal impairment burden in long-standing diabetes. Circles indicate outlier values (observations lying more than 1.5 × interquartile range from the box).

To visually assess the distribution of kidney function across diabetes duration strata, a boxplot of estimated glomerular filtration rate (eGFR) was generated (Figure 1). As illustrated, patients with long-standing diabetes (≥15 years) exhibit a marked downward shift in eGFR distribution compared with the other duration groups. While eGFR values remain relatively preserved in the 0–4, 5–9, and 10–14-year strata, a substantial decline is observed in the ≥15-year group, with a lower median and narrower interquartile range. This graphical representation supports the statistically significant differences identified in the comparative analyses and highlights the progressive deterioration of renal function associated with prolonged diabetes exposure.

### 3.2. Linear Regression Analysis of eGFR and Diabetes Duration

To further examine the association between diabetes duration and kidney function, linear regression models were constructed using estimated glomerular filtration rate (eGFR) as the dependent variable.

In unadjusted analysis, diabetes duration was significantly associated with declining renal function. Each additional year of diabetes was associated with a decrease of 1.32 mL/min/1.73 m^2^ in eGFR (β = −1.32; 95% CI −1.80 to −0.84; *p* < 0.001).

A multivariable linear regression model was subsequently performed, adjusting for age, sex, body mass index (BMI), hypertension status (HTA), and HbA1c. After adjustment, diabetes duration remained independently associated with lower eGFR, although the magnitude of the effect was attenuated. Each additional year of diabetes was associated with a 0.85 mL/min/1.73 m^2^ reduction in eGFR (adjusted β = −0.85; 95% CI −1.33 to −0.38; *p* < 0.001).

These findings indicate that diabetes duration exerts an independent and clinically meaningful effect on kidney function beyond the influence of age, hypertension, and metabolic factors. The persistence of statistical significance after multivariable adjustment suggests that prolonged diabetes exposure contributes directly to renal functional decline.

**Figure 2:**
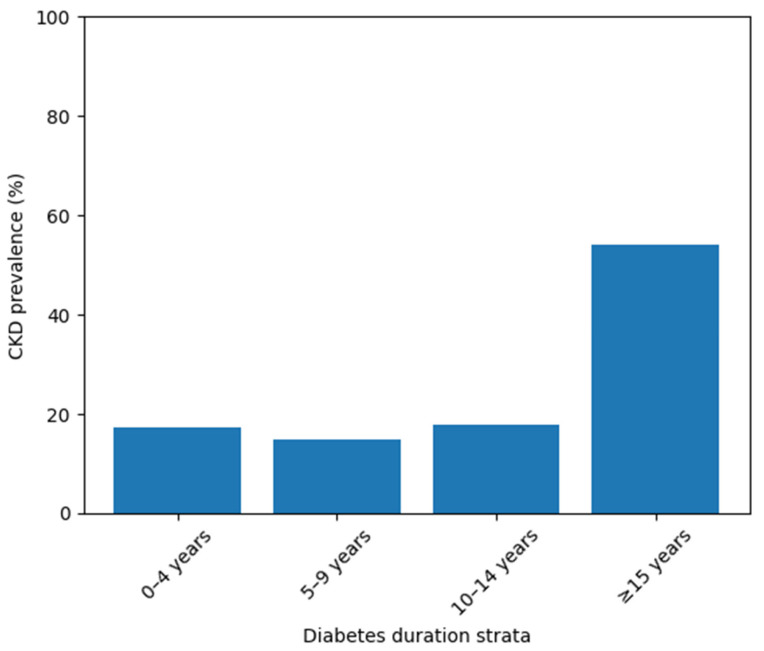
Prevalence of functional chronic kidney disease (CKD) across diabetes duration strata. Bar plot illustrating the proportion of patients with eGFR < 60 mL/min/1.73 m^2^ across increasing diabetes duration categories. A marked increase in CKD prevalence is observed in patients with ≥15 years of diabetes, consistent with a substantial renal burden in long-standing disease.

To further illustrate the burden of renal impairment across diabetes duration strata, the prevalence of functional chronic kidney disease (CKD; eGFR < 60 mL/min/1.73 m^2^) was analyzed and graphically represented (Figure 2). As shown, CKD prevalence remains relatively stable in the early and intermediate duration groups (0–14 years), ranging between approximately 15% and 18%. In contrast, a marked increase is observed in patients with ≥15 years of diabetes, where CKD prevalence exceeds 50%. This substantial rise supports the hypothesis of a duration-dependent escalation in renal vulnerability and visually reinforces the statistically significant differences identified in the comparative analyses.

### 3.3. Logistic Regression Analysis of Functional CKD and Diabetes Duration

To further evaluate the association between diabetes duration and functional chronic kidney disease (CKD), logistic regression models were constructed using eGFR < 60 mL/min/1.73 m^2^ as the dependent variable.

In unadjusted analysis, diabetes duration was significantly associated with increased odds of CKD. Each additional year of diabetes was associated with a 12.8% increase in the odds of having eGFR < 60 mL/min/1.73 m^2^ (OR = 1.13; 95% CI 1.07–1.19; *p* < 0.001).

After adjustment for age, sex, body mass index (BMI), hypertension status, and HbA1c, diabetes duration remained independently associated with CKD. Each additional year of diabetes was associated with a 10.7% increase in the odds of renal impairment (adjusted OR = 1.11; 95% CI 1.04–1.17; *p* < 0.001).

These findings demonstrate that prolonged diabetes exposure is independently associated with functional renal decline, even after controlling for major clinical confounders. Unlike the cardiovascular analysis, the association between diabetes duration and CKD persisted after multivariable adjustment, indicating a robust and independent relationship (Table 2).

**Figure 3:**
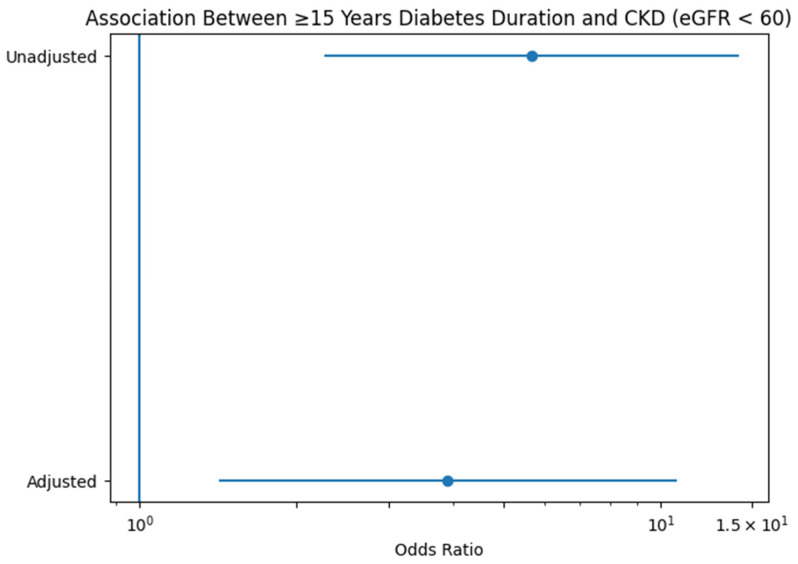
Association between diabetes duration and functional CKD. Forest plot illustrating unadjusted and adjusted odds ratios (OR) with 95% confidence intervals for the association between diabetes duration (per 1-year increase) and functional chronic kidney disease (eGFR < 60 mL/min/1.73 m^2^). The adjusted model includes age, sex, BMI, hypertension, and HbA1c. The vertical reference line indicates OR = 1.

To evaluate the magnitude of renal risk associated with long-standing diabetes, a forest plot was generated comparing patients with ≥15 years of disease duration to those with 0–4 years (reference category) (Figure 3). In the unadjusted model, long-standing diabetes was associated with a markedly increased odds of functional CKD, with an odds ratio exceeding fivefold. Although attenuation was observed after adjustment for age, sex, BMI, hypertension, and HbA1c, the association remained statistically significant, with nearly fourfold higher odds of CKD in the ≥15-year group. This visual representation highlights both the strength and robustness of the duration-dependent renal risk and supports the presence of a potential threshold effect after prolonged diabetes exposure.

Although the statistical models quantify the association between diabetes duration and renal impairment, the graphical representation provides an intuitive visualization of the magnitude and consistency of the effect across models and confidence intervals.

### 3.4. Sensitivity Analysis: Diabetes Duration per 5-Year Increase

To enhance clinical interpretability, diabetes duration was additionally modeled per 5-year increase in logistic regression analyses using functional CKD (eGFR < 60 mL/min/1.73 m^2^) as the dependent variable.

In unadjusted analysis, each 5-year increase in diabetes duration was associated with an 82% increase in the odds of CKD (OR = 1.82; 95% CI 1.39–2.39; *p* < 0.001).

After adjustment for age, sex, body mass index (BMI), hypertension status, and HbA1c, the association remained statistically significant. Each additional 5 years of diabetes was associated with a 66% increase in the odds of CKD (adjusted OR = 1.66; 95% CI 1.25–2.21; *p* < 0.001).

These findings confirm the robustness of the association between diabetes duration and renal impairment and demonstrate a clinically meaningful increase in CKD risk over relatively short temporal intervals (Table 3).

### 3.5. Categorical Analysis: ≥15 Years Versus 0–4 Years of Diabetes Duration

To further explore a potential threshold effect of prolonged diabetes exposure on renal impairment, a categorical logistic regression analysis was performed comparing patients with ≥15 years of diabetes to those with 0–4 years (reference group).

In unadjusted analysis, patients with ≥15 years of diabetes had significantly higher odds of functional CKD (eGFR < 60 mL/min/1.73 m^2^) compared with those in the early phase of the disease. The odds of CKD were more than fivefold higher in the long-duration group (OR = 5.65; 95% CI 2.26–14.11; *p* < 0.001).

After adjustment for age, sex, body mass index (BMI), hypertension status, and HbA1c, the association remained statistically significant. Patients with ≥15 years of diabetes had nearly fourfold higher odds of CKD compared with those with 0–4 years of disease (adjusted OR = 3.90; 95% CI 1.42–10.75; *p* = 0.008).

These findings demonstrate a strong and independent association between long-standing diabetes and renal impairment, supporting the presence of a clinically meaningful threshold effect after prolonged disease duration (Table 4).

**Figure 4:**
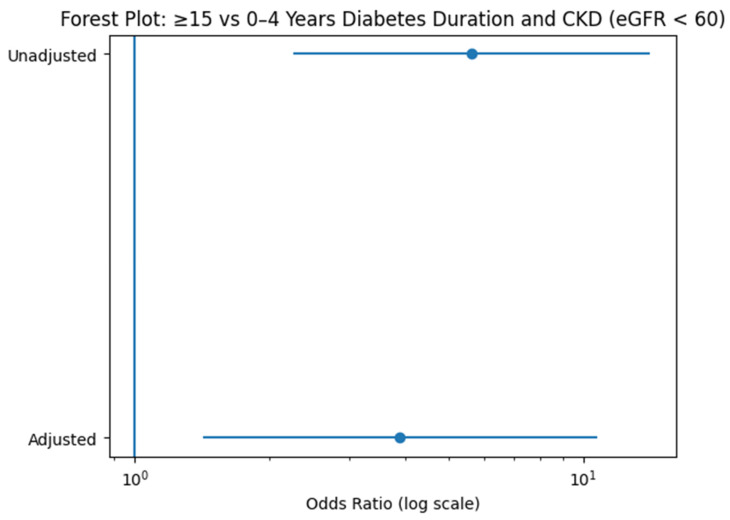
Forest plot of the association between long-standing diabetes (≥15 years) and functional CKD. Unadjusted and adjusted odds ratios (OR) with 95% confidence intervals for the comparison of ≥15 years versus 0–4 years of diabetes duration, using functional CKD (eGFR < 60 mL/min/1.73 m^2^) as the dependent variable. The adjusted model includes age, sex, BMI, hypertension, and HbA1c. The vertical reference line indicates OR = 1.

To further emphasize the magnitude and stability of the association between long-standing diabetes and renal impairment, a forest plot on a logarithmic scale was constructed (Figure 4). The log-scale representation allows clearer visualization of effect size and confidence intervals relative to the null value (OR = 1). As illustrated, patients with ≥15 years of diabetes exhibit substantially increased odds of functional CKD compared with those with 0–4 years of disease. Although adjustment for age, sex, BMI, hypertension, and HbA1c attenuated the effect size, the association remained statistically significant. This graphical presentation reinforces the presence of a strong and independent duration-related renal risk, consistent with a cumulative exposure model.

**Figure 5:**
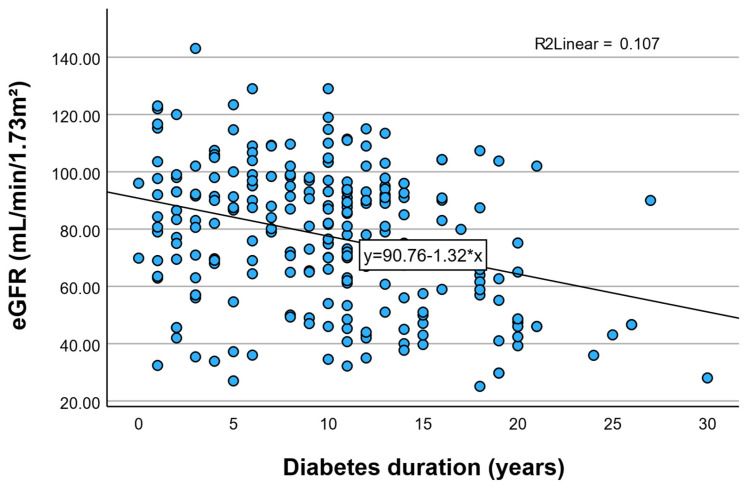
Association between diabetes duration and kidney function. Scatter plot illustrating the relationship between diabetes duration (years) and estimated glomerular filtration rate (eGFR, mL/min/1.73 m^2^). The superimposed linear regression line demonstrates a negative association, indicating progressive decline in renal function with increasing diabetes duration.

To further explore the continuous relationship between diabetes duration and renal function, a scatter plot with a superimposed linear regression line was generated (Figure 5). As illustrated, eGFR demonstrates a negative association with increasing diabetes duration, with an estimated decline of approximately 1.3 mL/min/1.73 m^2^ per additional year of disease. Although the coefficient of determination indicates a modest proportion of explained variance (R^2^ = 0.107), the overall trend is statistically significant and supports the concept of progressive renal decline associated with cumulative diabetes exposure. The dispersion of data points suggests inter-individual variability, yet the downward trajectory remains evident across the duration spectrum. The model explained approximately 10.7% of the variability in eGFR values (R^2^ = 0.107).

## 4. Discussion

In this clinical sample of adults with type 2 diabetes, diabetes duration was strongly and independently associated with declining kidney function. Both continuous and categorical analyses consistently demonstrated that prolonged disease exposure was linked to lower eGFR and substantially higher odds of functional chronic kidney disease (CKD). Unlike the cardiovascular analysis performed in the companion study, the association between diabetes duration and renal impairment remained robust after multivariable adjustment, indicating an independent effect of cumulative metabolic exposure on kidney function.

We observed a marked decline in eGFR across duration strata, with mean values decreasing from approximately 82–84 mL/min/1.73 m^2^ in early and intermediate disease to 61.6 mL/min/1.73 m^2^ in patients with ≥15 years of diabetes. Notably, more than half of patients in the ≥15-year group met criteria for functional CKD (54.2%), compared with approximately 15–18% in shorter-duration groups.

Linear regression analysis confirmed a significant decline of 1.32 mL/min/1.73 m^2^ per year in unadjusted analysis and 0.85 mL/min/1.73 m^2^ per year after adjustment for age, sex, BMI, hypertension, and HbA1c. Clinically, this corresponds to an estimated reduction of approximately 17 mL/min/1.73 m^2^ over 20 years of disease duration, which closely parallels the observed differences between early- and long-duration groups [17,18,19].

Importantly, when modeled per 5-year increment, diabetes duration was associated with a 66% increase in the adjusted odds of CKD, reinforcing the clinical relevance of cumulative exposure over relatively short time intervals [20].

Categorical analysis revealed a pronounced threshold effect. Patients with ≥15 years of diabetes had nearly fourfold higher adjusted odds of CKD compared with those with 0–4 years of disease. The magnitude of this association suggests that prolonged metabolic stress may accelerate structural and functional renal damage beyond a critical exposure window. This finding suggests that the burden of renal impairment increases substantially after prolonged exposure to diabetes, supporting the hypothesis that cumulative metabolic stress contributes to progressive kidney dysfunction.

This observation aligns with the concept of “metabolic memory,” whereby early and sustained hyperglycemia induces long-lasting epigenetic and microvascular alterations. Chronic exposure to hyperglycemia promotes glomerular hyperfiltration, mesangial expansion, basement membrane thickening, and progressive nephron loss. Over time, these mechanisms culminate in an irreversible decline in glomerular filtration [8,21,22,23,24].

A particularly important finding of this study is that the association between diabetes duration and CKD persisted after adjustment for age and hypertension—two major determinants of renal impairment. This suggests that cumulative diabetes exposure exerts a direct nephrotoxic effect beyond the expected decline associated with physiological aging or blood pressure–related damage.

This contrasts with the cardiovascular findings in the parallel analysis, where age largely attenuated the association between diabetes duration and ASCVD. The divergent patterns may reflect organ-specific vulnerability: while vascular disease is strongly influenced by systemic aging processes, renal function may be more directly affected by chronic glycemic injury and microvascular damage [25,26,27,28,29].

These findings underscore the importance of early and sustained nephroprotective strategies in patients with T2DM. The steep increase in CKD prevalence after 15 years of diabetes highlights the need for intensified monitoring and early implementation of kidney-protective therapies, including renin–angiotensin system inhibitors and SGLT2 inhibitors.

Furthermore, the progressive nature of renal decline demonstrated here supports proactive risk stratification based on disease duration. Even in the absence of advanced age, prolonged diabetes exposure should alert clinicians to elevated CKD risk [30,31,32].

This study benefits from a well-characterized real-world sample and the use of complementary statistical approaches, including continuous modeling, categorical comparisons, and sensitivity analysis per 5-year increment. The consistency of findings across models strengthens the internal validity of the observed association.

However, several limitations should be acknowledged. The cross-sectional design precludes causal inference and does not allow assessment of temporal trajectories or incident CKD. Albuminuria data were not included in the present analysis, limiting evaluation of early diabetic nephropathy stages. Additionally, residual confounding cannot be excluded despite multivariable adjustment.

Information on specific antidiabetic medications, including SGLT2 inhibitors or GLP-1 receptor agonists, was not incorporated into the statistical models. These therapies may exert nephroprotective effects and could partially influence renal outcomes.

Albuminuria measurements were not consistently available for all participants and were therefore not included in the present analysis. As a result, early stages of diabetic kidney disease, characterized by preserved eGFR but increased albuminuria, could not be evaluated.

Future longitudinal studies incorporating repeated eGFR measurements and albuminuria assessment would provide further insight into the dynamics of renal decline in long-standing diabetes.

Collectively, these findings demonstrate a strong and independent association between diabetes duration and renal impairment. Unlike cardiovascular burden, which appeared largely influenced by aging, kidney function decline showed a robust relationship with cumulative diabetes exposure. These results support the concept that prolonged metabolic stress exerts a direct and progressive impact on renal function, emphasizing the importance of early nephroprotective intervention in patients with type 2 diabetes.

## 5. Conclusions

In this clinical cohort of adults with type 2 diabetes, diabetes duration was strongly and independently associated with declining kidney function. Longer disease duration was linked to progressively lower eGFR values and substantially higher odds of functional chronic kidney disease, even after adjustment for age, hypertension, and metabolic factors.

Each additional year of diabetes was associated with a clinically meaningful reduction in renal function, and every 5-year increase significantly amplified the risk of CKD. Notably, patients with ≥15 years of diabetes exhibited nearly fourfold higher adjusted odds of renal impairment compared with those in the early phase of the disease, supporting the presence of a threshold effect after prolonged metabolic exposure.

Unlike cardiovascular burden, which appeared largely influenced by chronological aging in the companion analysis, renal dysfunction demonstrated a robust and independent relationship with cumulative diabetes duration. These findings emphasize the progressive nephrotoxic impact of sustained hyperglycemic exposure and highlight the need for early and continuous nephroprotective strategies in patients with type 2 diabetes.

Prospective longitudinal studies are warranted to further elucidate the temporal dynamics of renal decline and to determine whether aggressive early intervention can mitigate the cumulative burden associated with long-standing diabetes.

## Figures and Tables

**Table 1:** Kidney function parameters according to diabetes duration.

Duration Group	*n*	Mean eGFR ± SD	Median (IQR)	CKD Cases (*n*)	CKD (%)
0–4 years	52	82.45 ± 24.54	83.17 (68.75–98.00)	9	17.3
5–9 years	54	84.27 ± 22.34	87.76 (71.04–98.36)	8	14.8
10–14 years	96	78.72 ± 21.46	80.99 (67.86–93.02)	17	17.7
≥15 years	48	61.57 ± 22.38	58.15 (46.00–74.43)	26	54.2

eGFR—estimated glomerular filtration rate, CKD—chronic kidney disease (defined as eGFR < 60 mL/min/1.73 m^2^), IQR—interquartile range. Differences in mean eGFR across duration strata were assessed using one-way ANOVA, while differences in CKD prevalence were evaluated using the chi-square test (*p* < 0.001 for both analyses).

**Table 2:** Logistic regression analysis of functional CKD (eGFR < 60 mL/min/1.73 m^2^) and diabetes duration.

Model	Exposure	OR	95% CI	*p*-Value
Unadjusted	Per 1-year increase	1.13	1.07–1.19	<0.001
Adjusted *	Per 1-year increase	1.11	1.04–1.17	<0.001

CKD, chronic kidney disease; eGFR, estimated glomerular filtration rate; OR, odds ratio; CI, confidence interval; BMI, body mass index; HbA1c, glycated hemoglobin. CKD was defined as eGFR < 60 mL/min/1.73 m^2^. Odds ratios represent the change in odds of functional CKD per 1-year increase in diabetes duration. The adjusted model included age, sex, BMI, hypertension status, and HbA1c as covariates. * Adjusted for age, sex, BMI, hypertension status, and HbA1c.

**Table 3:** Logistic regression analysis of functional CKD per 5-year increase in diabetes duration.

Model	Exposure	OR	95% CI	*p*-Value
Unadjusted	Per 5-year increase	1.82	1.39–2.39	<0.001
Adjusted *	Per 5-year increase	1.66	1.25–2.21	<0.001

CKD, chronic kidney disease; eGFR, estimated glomerular filtration rate; OR, odds ratio; CI, confidence interval; BMI, body mass index; HbA1c, glycated hemoglobin. CKD was defined as eGFR < 60 mL/min/1.73 m^2^. Odds ratios represent the change in odds of functional CKD per 5-year increase in diabetes duration. The adjusted model included age, sex, BMI, hypertension status, and HbA1c as covariates. * Adjusted for age, sex, BMI, hypertension, and HbA1c.

**Table 4:** Logistic regression analysis of functional CKD: ≥15 years versus 0–4 years of diabetes duration.

Model	Comparison	OR	95% CI	*p*-Value
Unadjusted	≥15 vs 0–4 years	5.65	2.26–14.11	<0.001
Adjusted *	≥15 vs 0–4 years	3.90	1.42–10.75	0.008

CKD, chronic kidney disease; eGFR, estimated glomerular filtration rate; OR, odds ratio; CI, confidence interval; BMI, body mass index; HbA1c, glycated hemoglobin. CKD was defined as eGFR < 60 mL/min/1.73 m^2^. Odds ratios represent the relative odds of functional CKD in patients with ≥15 years of diabetes compared with those with 0–4 years (reference category). The adjusted model included age, sex, BMI, hypertension status, and HbA1c as covariates. * Adjusted for age, sex, BMI, hypertension, and HbA1c.

## Data Availability

The original contributions presented in this study are included in the article. Further inquiries can be directed to the corresponding authors.

## Abbreviations

The following abbreviations are used in this manuscript:

ASCVD	Atherosclerotic cardiovascular disease
BMI	Body mass index
CI	Confidence interval
CKD	Chronic kidney disease
CRP	C-reactive protein
eGFR	Estimated glomerular filtration rate
ESR	Erythrocyte sedimentation rate
HbA1c	Glycated hemoglobin
IQR	Interquartile range
RFG	Renal filtration rate (estimated glomerular filtration rate)
TRIG/HDL	Triglyceride-to-high-density lipoprotein cholesterol ratio
T2DM	Type 2 diabetes mellitus
